# Hospital Practice

**Published:** 1880-03

**Authors:** 


### HOSPITAL PRACTICE.

*St. Georges's Hospital.—Case of Hypospadias. (Under the care of Mr. T. Pickering Pick.)*

Plastic surgery, or "restorative surgery" as it is sometimes called, is always a subject of great interest, and one which merits the careful consideration of every practical surgeon. Operations performed for the cure of deformities, the relief of a hare-lip, the restoration of a nose, the closure of a fistulous opening, and such like, form a class in themselves, and require special attention and care on the part of the operator, if he would bring his case to a successful issue. At the same time there are perhaps no cases which are so satisfactory when successful, and which bring greater credit to the surgeon. Deformities are always of a distressing nature to the patient, and entail a great amount of suffering, either bodily or mental, and any operations which can be performed for their relief are sought eagerly after and gladly welcomed. The following case of hypospadias is recorded as affording a good example of what may be achieved in a form of malformation not unfrequently met with, and which, though it does not entail much bodily suffering, is the cause of very considerable mental distress to the patient.

Henry B——, aged thirteen, was admitted on Jan. 19th, 1875, suffering from hypospadias. The penis at first sight appeared to be quite rudimentary, consisting merely of the glans, which, however, was well developed and was covered on its dorsal surface by a considerable semilunar fold of skin and cellular tissue, which formed a sort of hood to it. This was the prepuce, malformed by being cleft on its under surface, heaped up on the dorsal aspect of the glans into a semilunar fold, and attached by its extremities to the scrotum on either side of the rudimentary penis. Upon examining more closely, it was found that the body of the penis was bound down to the scrotum by a broad fold or web of skin. When erection took place it was arched, with the convexity upwards. The orifice of the urethra was in the scrotum, at the anterior extremity of the median raphé, and was situated at the bottom of a sort of fossa or cul-de-sac in this situation. Just above it was the glans penis, overhanging the orifice, and in the center of the glans was a slight depression in the normal position of the meatus urinarius. The boy was a fine-grown, healthy, intelligent lad, and presented no other malformation.

The first operation (Feb. 10th) consisted in making a traverse incision through the whole thickness of the semilunar prepuce just in front of its attachment to the corona glandis, so that it was only attached to the sides of the scrotum and penis by its two extremities. The mucous membrane at the margins of the incision was then stitched to the corresponding skin on either side, and the glans penis was protruded through the button-hole opening which was thus made. By this proceeding the malformed prepuce was brought down beneath the glans penis, and formed a sort of bridge stretching across from one side of the organ to the other, while the glans protruded through the opening thus made. The wound took some time to heal, but eventually did so, and a passage then existed between the inverted prepuce or bridge of skin and the under surface of the glans penis, which was intended to form part of the urethra.

The second step of the operation (May 12th) was undertaken to liberate the body of the penis from the scrotum. This was done by dividing the web of skin which connected the two together, and thus gradually raising the penis. The result of this was a large diamond-shaped raw surface where the one organ had been separated from the other. The edges of this surface were drawn together by hare-lip pins, so as to convert it into a longitudinal wound. In performing this operation care was taken not to interfere with the points of attachment of the bridge of skin formed by the inverted prepuce, by making the incision a little posterior to it, for fear that its vitality might be destroyed

After the wound had healed, it was found that there was a fairly formed penis, about an inch in length. At the anterior extremity of this was the glans, with the inverted prepuce on its under surface. On the anterior surface of the scrotum, some distance below the point of attachment of the skin of the scrotum to that of the penis, was the opening of urethra. The space between these two points—that is to say, the glans penis and the opening in the scrotum—was occupied by the cicatricial tissue which was the result of the previous operation, and was intended to form the roof of the new urethra.

The third step (June the 26th) consisted in bringing over two oblong flaps to form the floor of the urethra, by a somewhat similar operation to that which is described and advocated by Mr. Holmes for extroversion of the bladder.* On the left side an oblong flap, with its base towards the middle line, was dissected up from the tissues on the sides of the scrotum and penis, so that its attached border was some little distance to the left of the middle line just external to the cicatricial tissue before mentioned. This flap was turned over, like the leaf of a book, so that the skin surface was directed towards the opening in the scrotum, and was intended to form the floor of the urethra, and the raw surface turned outwards. On the right side an oblong flap was fashioned, one side of the flap skirting the right border of the cul-de-sac or fossa in which the urethra was situated, and being carried upwards and to the right side of the penis; a second incision was made parallel to this, and about an inch external to it, and the two joined by a cross incision at the lower extremities, so that the attached border was directed upwards and outwards. This flap, having been dissected upwards, was applied to the raw surface of the left flap by gently sliding it round, in the manner described in the operation for extroverted bladder, and the two flaps were united together by several points of silver suture. The wounds caused by the transplantation of the flaps were also closed by suture. Union took place very readily, and we had now a second bridge of skin which covered over the linear cicatrix in the middle line, which had been formed as the result of the operation for

---

* Surgical Diseases of Children, page 149.

separating the penis from the scrotum. This bridge extended from the opening of the urethra below, almost to the glans penis above. The next step (July 10th) consisted in attaching the lower margin of this bridge to the skin of the scrotum below the opening of the urethra. This was done by rawing the lower border of the flap and implanting it into a transverse groove which was made in the front of the scrotum, just below the fossa, by slightly dissecting up the skin, so that the flap was dovetailed, as it were, into the skin of the scrotum, and fixed there by silver sutures, and thus the opening into the cul-de-sac from below was closed.

This operation was not entirely successful, a small fistulous opening being left at one point, but this was subsequently closed July 24th, and the urine now flowed over the flap or bridge, and was discharged above its upper margin. The final step in the proceeding consisted in attaching the upper border of this bridge to the lower border of the inverted prepuce. This was done by rawing the two margins, and sewing them together with several points of silver suture. During the attempt to close this opening the water was drawn off, and the patient was not allowed to pass any of himself, because it was feared that some of the urine might find its way between the edges of the wound and interfere with union. The result, however, was most satisfactory; complete union took place, throughout the entire length of the wound. The series of operations were now completed, and a sort of canal had been formed which extended from the opening of the urethra in the front of the scrotum to the base of the glans penis, which it was hoped would be sufficiently perfect to enable the patient to perform the act of copulation with the result of ejecting the semen into the vaginia, a proceeding which would clearly have been impossible previous to the performance of the operation.

Four years have now elapsed since the case was completed, and the following is the condition of parts: The boy is just eighteen, but does not look more than sixteen. The penis is small, but otherwise well developed. On the under surface of the organ is a canal, which has been formed as above described, which extends, from the corona glandis to the original orifice of the urethra in the scrotum.

This canal is rather large, so much so, in fact, that it would be possible, with a certain amount of stretching, to introduce the little finger. It is about an inch and a half in length, and by dilating its orifice one can see at the bottom the opening of the urethra on the anterior wall of the scrotum. There can be little doubt from the condition of the parts that all the natural functions could be satisfactorily performed.

*West London Hospital.—Urethral Calculus Impacted behind a Tight Traumatic Stricture; Continuous Dilatation; Spontaneous Expulsion of the Stone; Patient attacked with Pleurisy when about to return home Cured; Death. (Under the care of Mr. Teevan.)*

George O——, a laborer, forty years old, was admitted into the hospital on Jan. 7th, 1879, having been put under Mr. Teevan's care by Dr. Hurdwood, of Leatherhead. From notes taken by Dr. Warrilow, the house-surgeon, it appeared that the man was formerly a soldier, and had suffered from fever in India and from ague in the Mauritius. In 1865, whilst engaged with some soldiers at St. Helena in hoisting a gun from a boat into a ship, the gear broke. Several of the men were killed, and the patient fell astride the gunwale of the boat. Blood escaped from the penis, and a swelling formed in the perineum. He was taken into the military hospital, where an incision was made into his perineum, much blood and urine flowing out. For three years he had been troubled with difficulty in making water, for which he sought advice, and had the stricture dilated to No. 12 E. On several occasions he had rigors, and suffered from pain in the loins. Two weeks ago no instrument could be passed.

When admitted to the hospital he was thin and sallow, had difficulty in micturition, and his urine contained some muco-pus. The scrotum and perineum were scarred with many cicatrices. No catheter could be introduced through the stricture, which commenced just in front of the bulb. An olivary whalebone bougie was wriggled through the contraction, and struck what was apparently a calculus, which could be felt through the rectum or perineum. With a little trouble the bougie was worked past the obstruction into the bladder, and there retained.

On January 9th the instrument was removed, and two hours afterward the patient expelled a phosphatic calculus about an inch long, resembling a piece of the stem of a common clay smoking-pipe. The stricture was gradually dilated, an olivary elastic catheter being passed every other day. During the whole of the period the patient was up and about the ward giving a helping hand.

On January 28th Mr. Teevan introduced No. 20 F., and, as the patient was able to pass the catheter for himself, he arranged to return home in a few days.

On the 30th Mr. Warrilow introduced No. 20.

On the 31st, 9 a. m., patient stayed in bed, complaining of headache and pain in the limbs. Pulse 100 ; temperature 99.8°. Temperature 100° at 8 p.m.

On February 1st he was more indisposed. Temperature 103° ; pulse 120. In the evening he suffered much from dyspnœa and paroxysmal pain in the left hyprochondriac region. Had a subcutaneous injection of morphia, which relieved the pain, and an enema, which removed the constipation and tympanites. The abdomen was covered with a linseed-meal poultice. Ordered brandy, milk and ice.

February 2d.—9 a. m.: Great pain in region of heart. Countenance anxious. Knees drawn up. Abdominal tenderness in left hypochondriac region. Pulse 99; temperature 120°. At 5 p. m. the patient commenced vomiting a clear fluid at intervals. Tongue covered with a thick yellow fur. Body bedewed with a cold clammy sweat. Vomiting ceased at 11 p. m. Ordered nutrient enemata and morphia.

3d.—Vomiting recommenced at 5 a. m., and continued throughout the day. The patient was examined by Dr. Fish, under whose care he was now placed. Dullness over left lung in axillary region ; vocal fremitis diminished ; shrill tubular note. Dr. Fish pronounced the pain to be pleuritic. Linseed-meal poultices were applied over the back and side, the nutrient enemata were discontinued, and the patient was ordered brandy and milk. Temperature 103°; pulse 130.—10 p. m.: Temperature 100°; pulse 120.

4th.—9 a. m.: Temperature 100.2°; pulse 99. Delirious part of the night, but obtained some sleep in the early morning. Free from pain. Bowels open.

5th.—9 a. m.: Temperature 99.2°; pulse 132. No vomiting. Respiration more hurried. Takes ten ounces of brandy.

6th.—Slept well. Decided improvement. Tongue clean. Bowels open; motions solid. Temperature 99.2°. 8 p. m.: Temperature 100°.

7th.—Physical signs much the same. Takes nourishment freely. Temperature 99.2°. 8 p. m.: Temperature 100.8°.

8th.—Temperature 99.4°; less dullness on percussion; less pain. 8 p. m.: Temperature 100°.

9th.—Temperature 99°; face pinched and dusky; breathing hurried; respiration 26; suffers but little pain. 8 p. m.: Temperature 99.8°.

10th.—Temperature 99.2°; restless and delirious in the night; takes but little nourishment. 8 p. m.: Temperature 99°; more pain in side; bulging in intercostal spaces; more dullness on percussion; had severe paroxysms of dyspnœa at night.

11th.—Had a restless night; great pain in side; more bulging in intercostal spaces; area of dullness greater. About 8:30 a. m. the patient asked to be raised in bed, when he suddenly fell back asphyxiated.

*Necropsy*, Feb. 13th, 10 a. m.—Chest: Left pleural cavity contained upwards of a quart of muco-purulent fluid; left lung compressed and flattened, weighed only seven ounces and a half; pieces of it sank in water. Right lung weighed twenty-two ounces and a half, and contained a few tubercles. Heart healthy. Abdomen: Spleen and liver healthy; left kidney seven ounces, right one six ounces; they were both examined microscopically by Mr. Augustus Pepper, who pronounced them healthy. The bladder and prostate were healthy; there were two false passages extending into the substance of the gland for a short way, which were lined with a mucous membrane, and were evidently of considerable age. In the urethra the remains of the stricture could be seen, and it was evident that the stone, which was phosphatic, had been formed in the dilated portion of the canal behind the contraction.

*Remarks by Mr. Teevan.*—The patient when quite cured of his local ailment, and about to return home cured, was attacked by pleurisy. Unfortunately the man's constitution had been deteriorated by the fever and ague from which he had suffered whilst in the tropics, so that he was predisposed to catch cold, and formed one of the many victims to the excessively low temperature which prevailed at the time of his illness. Happily, in the present instance, no operation had been performed, so that surgery must be held blameless for the unfortunate termination. When the patient was admitted he was not suffering from retention, and was able to pass urine by the side of the calculus. The case illustrated what could be achieved by apparently so insignificant an instrument as a fine whalebone bougie, which in the present instance had effected the dilatation of the stricture and the expulsion of the stone.

*Somerset Hospital Cape Town, Cape of Good Hope.—Inguinal Aneurism; Ligature of the Right Common Iliac Artery; Recovery. (Under the care of Dr. A. L. Chiappini).*

The following notes were taken by the late Dr. W. H. Wood:

W. H——, forty, was admitted on January 2d, suffering from a very large inguinal aneurism, which could be distinctly seen pulsating through his trowsers, and forming a prominence on the right side. He was of intemperate habits, but had never had syphilis. He stated that about fifteen months before admission he was engaged in loading a vessel with copper ore at night, and that when lifting a particularly heavy bag he felt something give way on his right side, and he immediately had very severe pain in the right groin, and about three hours after this, on putting his hand within his trousers-pocket, he felt a lump, which was not very hard, and about the size of a pigeon's egg. At nine next morning he showed it to a medical man, who ordered him to poultice the swelling, which was now very hard. He lay up and poulticed for nearly a week, when, the pain having diminished, he returned to work, and two months after again showed it to the same gentleman. The tumor was now the size of a hen's egg, and the man noticed for the first time that it pulsated. He was told to continue the poultices. Time passed on, the man being at work on and off; the tumor still increasing and causing great pain down the right leg. About three months before admission the "lump" was, he said, about the size of a "baby's head," and he again showed it to the doctor, who advised him to poultice continually. A few weeks before admission he saw another medical man, who immediately ordered him to the hospital, to which he went by sea.

On examination, an immense pyramidal tumor was seen, pulsating with a strong expanding impulse, occupying the whole of the right iliac region, being raised about five inches above the surface; a tape lay across it measuring just a fraction under twelve inches from edge to edge. The upper limit was level with the crest of the ilium; to the left it approached closely to the pubes, while it was prolonged downwards into the thigh, Poupart's ligament being tightly stretched over its lower portion. The tumor was soft and elastic; at the apex the sac was very thin, and the skin a dusky purple, and it was evident it mus-soon give way. The right leg was considerably swollen; the temperature was the same in both legs.

On the 8th, or six days after admission, the patch at the apex was larger and thinner, and after consultation it was decided to operate the next day.

On the 9th Dr. A. L. Chiappini, the visiting surgeon, accordtingly proceeded to operate, making a semilunar (from the small space at his disposal, almost a semicircular) incision, with its convexity at the furthest about five inches from the umbilicus. The peritoneum, which was not adherent, was pushed back with the intestines, and the common iliac artery exposed (as was expected, there was nothing to be seen of the external iliac artery) and ligatured with thick silk. One end of the ligature was left hanging out, and carbolic dressings were applied to the wound, which was brought together by a few sutures. He was then put to bed, and the leg, previously shrouded in cotton wool, was surrounded with hot sand bags and hot-water bottles. In the evening his pulse was 90; he had not much pain anywhere. Had half a grain of morphia. Had a good night and passed water freely. The next morning the tongue was clean; pulse 112; temperature of body 99.5°. At 9 a. m. the pulse was 100; the temperature 99°. Repeat the sedative draught.

He continued without one bad symptom, not suffering so much from pain and numbness in the leg as before the operation, and having perfect sensation in the leg. The tumor very rapidly decreased, at one time, however, threatening to suppurate, and on the 20th day after the operation the ligature came away. The man is now in perfect health, and working as a navvy on the railroad works.

*St. Bartholomew's Hospital.—Three Cases of Chronic Meningitis, Following Injury to the Skull. (Under the care of Dr. Southey.)*

Severe localized headache, delusions, mental aberration, signs of paralysis, epileptic fits, and other symptoms as sequelæ of injuries of the skull, are more familiar to surgeons than to physicians. During the present year we have published in the *Mirror* many cases of fracture or other injury to the skull followed immediately, or after a longer or shorter interval, by symptoms of cerebral lesions or mental impairment. We may indicate some of the cases included in the instructive series taken from Dr. Hulke's clinique at the Middlesex Hospital, and distributed over many issues; two cases reported from Mr. Jonathan Hutchinson's wards in the London Hospital; and the extremely interesting cases that were under Mr. Davis-Colley's care in the Seamen's Hospital, Greenwich.

The following cases will, in addition to their own intrinsic merits, give increased importance to those just referred to. They show that chronic meningitis may follow blows on the head at a period so remote that the connection between the two may be entirely overlooked or misinterpreted. As Dr. Southey pointed out in some comments on these cases, the difficulty of tracing any connection between the injury and the symptoms of meningitis is usually increased by the circumstance that when the patients come under observation they are often unable to give any trustworthy account of themselves. The symptoms may point distinctly towards chronic meningitis, but the initial lesion is not made known. The presence of tuberculosis is too often accepted as an ever-ready explanation of the phenomena; whereas these cases of late traumatic meningitis are wholly unlike, in course, duration and morbid anatomy, tubercular meningitis, though simulating it in their clinical characters.

CASE I. *Chronic Cerebral and Spinal Meningitis occurring Four Years after a Fall from a Window; Death.*—Mary Ann S., aged twenty-seven, a pale but fairly-nourished woman, was admitted Nov. 5, 1878, with vomiting and diarrhœa. Her appearance was not prepossessing, in consequence of a deep indentation of the bridge of the nose, the result of an old fracture. The mental state was peculiar. She could give no account of her illness; but she possessed all the special senses, as well as power of motion and of sensation, and a little, though degraded, intellectual power. Though listless, morose and indifferent to questions directly addressed to her, she every now and then threw a remark into her mother's statement respecting her ailment, confirming, correcting, or repudiating it indiscriminately. Her memory was obviously defective, but there was an apparent desire not to mislead or to have her history misstated.

The remains of the old fracture of the nose suggested an inquiry into the circumstances of the accident. It was alleged that four years previously the patient fell out of a window (the height was not ascertained), and was picked up unconscious and bleeding from the nose. Consciousness soon returned, and beyond the depression of the bridge of the nose, she was not thought to have suffered any particular damage. She set about her usual domestic duties at once, and made no further complaint. About two years after this she married, and was confined of a healthy child about eighteen months afterwards. She was suckling the child, aged six months, at the time of admission, and had a moderate amount of milk in both breasts. After her confinement she remained low-spirited and ailing, and five weeks before admisson her husband and relations noticed her conduct to be strange; she talked a good deal of nonsense, they said, occasionally wandered about the house at night, and was unable to give any good reason for her vagaries. About a fortnight before admission her hands became red, swollen and painful, so that she could hardly move her fingers. This was attributed to rheumatism. She gradually became more delirious and difficult to restrain or keep in bed, and three days before entering the hospital vomiting supervened. She was stated to have been healthy, of temperate habits, industrious and happy in home.

When taken into the hospital she walked up stairs slowly and stiffly, and required little help to undress herself. She seemed stiff and weak, and moved her head stiffly, and she complained of pain down the spine, especially at the cervical portion. There was, however, no tenderness on pressure over any of the spinous processes. The pupils were equally dilated, and reacted slowly to light. The physical signs of heart, lungs and abdominal viscera were normal. The tongue was thickly furred and indented at the edges. The bowels were confined. Temperature 99.4°; pulse feeble, 92; respiration 23, tranquil and regular. At first she refused to take some beef-tea, saying she should only bring it up again. After a little pressing she did take some, and at once ejected it. Urine: thirty to forty ounces passed daily, acid; sp. gr. 1030; yielding a deposit of urates, and containing a trace of albumen, but no sugar.

The diagnosis was chronic cerebral and spinal meningitis, but whether of tubucular nature or not was left undecided.

She was fed frequently with small quantities of milk and beef-tea, and had nutrient enemata whenever the stomach rejected food. She was quieted with draughts of chloral hydrate and bromide of potassium combined; occasionally she needed hypodermics of morphia. She had alterations of excitement and depression. On some nights her cheeks would be flushed, and she was noisy and troublesome and called out violently; and then would follow fits of extreme depression, in which she was completely indifferent to what was done for her, perverse, willful and apparently conscious of the trouble she was giving, but choosing to pass her urine and fæces in bed. At the professional visits she could rouse herself and answer questions rationally, though not always correctly; afterwards she would ramble and mutter incoherently. On one occasion she cried when asked about her baby; on another she denied ever having had one.

On Nov. 9th and 10th the temperature rose to 101° and 101.8°, and on the 11th it ranged between 98.4° and 101.8°. It never reached 102°.

Rarely she enjoyed her food, and went twenty-four hours without vomiting. By ophthalmoscopic examination, the left optic papilla was found to be redder than natural, and the veins were rather swollen, but there was no effusion.

Towards the end of November the periods of mental excitement became fewer, and she remained perfectly passive, absolutely demented. On the 28th she was violently convulsed; the fit lasted half an hour, and was succeeded by rigidity of the legs which did not pass off for some hours. During the seizure the pupils widely dilated. A comatose sleep followed, and on awakening she was more rational, and for several days after there was less vomiting. Urine, sp. gr. 1032, with traces of bile and albumen, but no sugar.

On Dec. 2d she lay huddled up in bed like an ordinary case of dementia, quite quiet, and appearing to suffer pain in the neck when moved. Pupils dilated, face flushed; pulse very feeble, regular; temperature normal. The right optic papilla was hyperæmic; the left was ill-defined at the margin, and the adjacent retina was hazy. There was also some disturbance of the choroidal pigment.

On Dec. 8th at 3 p.m., she had a severe rigor, during which the axillary temperature was 98.5°; pulse 136. No reactionary fever followed. She daily grew feebler, but did not exhibit any signs of paralysis, and could turn herself in bed up to the last. She died quite suddenly on Dec. 16th.

*Necropsy* (Dec. 17th).—Thoracic and abdominal viscera all healthy, except the liver, which was slightly fatty, and the gall bladder contained four gall-stones. Head: The skull-cap was somewhat adherent to the dura mater about the Pacchionian glands and in one or two other places. The pia mater over the base of the brain was thickened and opaque, and the veins were swollen. The convolutions of the hemispheres were flattened. The pia mater, lining each side of the Sylvian fissure, on both sides was adherent, as was also the adjoining surfaces of the two cerebral hemispheres, preventing a proper view of the corpus callosum, without forcible separation. The lateral ventricles, the third ventricle, and the aqueduct of Sylvius were all widely dilated, and contained a slightly turbid serous fluid. The choroid plexuses were collapsed. The lining membranes of the ventricles were opaque, the opacity extending down into the fourth ventricle, and then to the space between the peduncles of the crebrum. At this spot, a dense opaque membrane, continuous with the pia mater, abruptly closed the passage, so that the intra-ventricular fluid was cut off from its normal communication with the spinal arachnoid channel. Thus a false membrane, the product of chronic meningeal inflammation, extended from the posterior part of the median lobe of the cerebellum to the posterior pyramids of the medulla oblongata, and blocked the natural passage by which the cerebral and spinal subarachnoid fluids intercommunicate. As a result of this, the ventricles of the brain were dropsically distended, and the convolutions flattened from compression. No tubercle or appearances at all resembling it were discoverable on the pia mater, or on the walls of the smaller arteries.

The brain is preserved in the hospital museum.

The question is, Did the chronic meningitis depend upon and originate from the injury received at the fall four years before?

CASE II. *Fall on the Back of the Head, followed Four Months after, by Cerebral Symptoms; Death.*—John A., aged sixteen, a fairly nourished, but pale, lymphatic lad, with a vacant, half-imbecile expression, was taken to the hospital by his mother on July 10th last. He cried and sobbed in a frightened, child-like manner, and seemed intellectually degraded. His skin felt hot, dry and desquamating; his tongue was moist and a little furred; pulse 108, regular and small; respiration about 20. The temperature would be normal or below normal in the morning, but suddenly rise to 102° between 2 and 8 p. m. He walked up stairs, but stood like a person weak in his knees, and with his legs somewhat wide apart. He required help to undress himself, but not because of any special paralysis. The only muscle in which any impairment of power eould be discovered was the external rectus of the left eye. After he was in bed he assumed the crouching attitude, lying habitually on his left side, with his knees drawn up and his hands between his thighs, as if always feeling cold.

The physical examination of the heart, lungs and abdominal organs elicited no facts indicative of disease.

The patient had enjoyed perfect health, and intellectually had been equal to boys of his own age, up to Christmas last (1878-79), when, while sliding on the ice, he had fallen down and struck the back of his head heavily, suffering some concussion of the brain, and was laid up in bed at home for a week after the accident. He resumed his employment, however, and was able to do his work as well as before his fall up to May 1st. About this date he had a bad attack of vomiting, and since that vomiting the lad seemed to his mother to have lost all his wits, because dreamy and apathetic, and able to remember nothing properly. From May to July, the date of his admission, he lay at home in bed most of the day, vomiting frequently, but still keeping a good deal of his food down. Latterly he had troubled his family very much at night-time, waking up, screaming violently, keeping everyone awake about him, sobbing like a naughty child, and refusing to be comforted or quieted. By day he was drowsy, apathetic, slow in responding to questions, but possessed of ordinary discernment; while at night he was restless, terrified and irrational. His bowels were inclined to be costive. He had no particular thirst, and he passed an average of three pints of urine per diem, sp. gr. 1020, containing no albumen, and showing no noticeable excess of earthy phosphates.

He was ordered tepid baths, with occasional cold spinal douches; a wet rag to his forehead when his temperature was high; a grain of calomel, followed by a saline, when the bowels had been confined for forty-eight hours; a nourishing diet, but no stimulant.

Observations in the hospital entirely confirmed the account received about him. On some nights he slept pretty quietly, and others he would scream and sob and disturb the ward. He behaved like a great baby at all times, but was clean in his habits. He did not complain of much pain in his head, or appear to suffer headache, but he often asked to have his head chopped off, and was at times possessed with the idea that it was desired to chop it off.

He took very little solid food, plenty of milk and fluids; as a natural consequence, he passed large quantities of urine, 70 to 115 ounce sometimes per diem. Scybala accumulated in his colon, and required clearing out artificially once. He only vomited once or twice in July; entertained a variety of delusions; thought his bed was inhabited first by rats then by eels, and that various animals, elephants, visited him. A restless noisy night was, however, often succeeded by vomiting on the following day.

On August 6th a careful ophthalmoscope examination was made by Mr. B. Vernon, who reported the right optic disc extremely red; left disc red, in parts ill-defined; retinal veins engorged.

About the end of August no more delusions were heard of. The temperature-chart was carefully kept from July 10th to Aug. 14th. For three days in succession the temperature would be maintained between 101.5° and 104°, then it would drop to normal or below (97.2°) for two days, and then rise again gradually each night one or two degrees until it reached 104.8°, or, as it did on one occasion, 105°. Latterly he complained more of headache; the temperature fluctuated less, and was seldom above 102° or below 99°; he became weaker muscularly; his tongue was tremulous, and although he could sit up in bed the effort to do so brought on intense pain in the head and vomiting. Tâches cérébrales were well marked.

Counter-irritation was applied over the occiput, and iodide of potassium was given internally, and chloral hydrate and bromide of potassium occasionally. In September recourse was had to hypodermics of morphia, with great relief to pain and vomiting. On September 15th it was determined to put a seton in the back of the neck. The first attempt was made with a silk thread, which was soon pulled out again. Two days later he was anæsthetized with chloroform, and a proper seton was introduced. He had scarcely recovered from the effects of the chloroform, four hours only having intervened, when he had the first of a series of epileptic fits, recurring at short intervals until death on the same evening. Death occurred ten weeks after his admission, or about twenty weeks after the manifestation of the first cerebral symptoms, and over nine months after the receipt of an injury to the head.

*Necropsy.*—The scalp and calvaria were healthy. There were many tough adhesions at the fork of the optic commissure, and a layer of lymph on the surface of the olfactory nerves and the adjacent cerebral hemispheres. The convolutions were flattened on both sides, and the whole base of the brain was covered with tough, but not thick, deposits of lymph. The sides of both Sylvian fissures were adherent, and the cerebral hemispheres were adherent in front. The fourth ventricle was closed by adhesions, and in each lateral ventricle three or four ounces of clear serum were found. The iter was closed, but seemed not quite impervious on dissection. The brain-substance was healthy; there was no tubercle anywhere. The spinal cord and membranes were also healthy. There was no tubercle or evidence of disease in the lungs or bronchial glands. The heart and the abdominal viscera were likewise free from evidences of disease.

The following is the third, and last, case of this interesting series:

Esther S——, aged twelve, an anæmic, scrofulous, ill-nourished child, was admitted into Faith ward on March 4th, 1875. She had a vacant expression, with a knitted frontal frown. She lay curled up in bed, refused all blandishments, and pulled the bed-clothes tightly round her whenever an attempt was made to examine her. She seemed deaf, would not answer questions, and was altogether morose. She walked into the ward, and on inquiry it came out that she had always been wayward and ill-tempered. About Christmas time (1874) she fell violently on the back of her head. No notice was taken of this, but some time afterwards she became dull of hearing and appeared deaf, although she sometimes undoubtedly heard what was said; she was thought to be shamming. There was great difficulty in getting her out of bed in a morning, indeed she generally had to be pulled out. Nine weeks before admission she was confined to her bed entirely, on account of constant headache, and of aching pain—supposed to be rheumatic—in the limbs and joints. She was capricious and restless, and often moaned or cried out during the night, or when left alone. Her bowels were constipated. Ever after the fall she was, her mother alleged, an altered child, though little notice was taken of the fall at the time.

On admission, the temperature was normal; pulse 96, irregular and feeble; respiration 20. The heart's action was irregular and slightly intermittent; the impulse was normal in situation, and there was no increase of præcordial dullness. There was good resonance over both lungs; the left supra and infra-clavicular regions were rather flat, and the breath sounds were feeble there. There was no evidence of any abdominal mischief. Lymphatic glands were everywhere natural to the touch. The urine was normal in quantity and character, but was chiefly passed in bed.

Except that she put out her tongue when asked to do so, there was scarcely any intelligent response to questions. At times she was very troublesome, and swore and made a great noise. The pupils were rather dilated, but there was no sign of paralysis of any of the ocular muscles. No ophthalmoscopic examination could be made, but it was evident from observations she occasionally made, that she could see.

She always fed with a spoon; on some days she would take a fair amount of nourishment and would be more awake, on other days she was so drowsy that she could not be got to swallow; she would even go to sleep with her mouth full of food, and had to be well shaken before she could be roused to masticate the food which perhaps was put into her mouth half an hour previously.

On March 12th she recognised her mother, and her face was flushed with excitement at the visit. Temperature 101.2°; pulse 84. The bowels had not been open for three days. She had vomited only once since admission. The eyelids were half closed and she groaned and occasionally screamed out.

On the 16th she stared about wildly, picked her nose, rubbed her forehead, pulled out her eyelashes, and moaned whenever she was disturbed. Pulse irregular, 108 to 120. After this she became gradually feebler and more soporous, and for the last few days of her life had to be fed by enemata. She died on March 21st, seventeen days after admission, or three months from the commencement of the symptoms.

At first a mixture containing ten grains of chloral hydrade and of bromide of potassium was given every six hours, but this was discontinued after the second day, after which the treatment became purely expectant.

*Autopsy* (March 22nd).—The pia mater and arachnoid were highly injected. There was some flattening of the convolutions from increase of the intra-ventricular fluid, four or five ounces of which escaped at the infundibulum just behind the optic commissure when the brain was removed. There was no appearance of effused lymph at the base of the brain, the substance of which was everywhere sound and healthy-looking. No tubercle was discoverable in the pia mater or fine vessels. The gray substance was, perhaps, rather pinker than natural, and a tiny blood-clot, the size of a pin's head, was found in the center of the pons Varolii. The olfactory bulbs were very thin. The other organs of the body were wasted, but otherwise healthy.

*Remarks.*—It would seem that in this case, too, some false membrane or adhesion prevented the free circulation of the sub-arachnoid fluid, and led to dropsical distension of the ventricles and consequent pressure upon the brain-substance. The alleged fall or injury may or may not have been the starting-point of subsequent changes. It was attended by serious concussion, and was passed over as a trifling circumstance. The original small intellectual endowment made it difficult to fix the exact date of the perverted cerebral functions.

*Middlesex Hospital.—Case of Fracture of the Base of the Skull (Under the care of Mr. Hulke.)*

The following report completes the series of cases of fracture of the skull.

CASE IV. *Extensive fracture of the base of the skull by a fall upon the street pavement, probably in an epileptic fit; bleeding and serous oozing from the ear; diffuse meningitis; death on the fifth day.*—The extent of the injury to the skull in this case is remarkable, occasioned as it presumably was by a simple fall upon the pavement—not from a height; for it can hardly be reasonably entertained that the man had been struck a violent blow on the head in the open street at midday without its having been witnessed by others; the motive of theft, too, was wanting, and his character was inoffensive and good. It was ascertained that he had been epileptic for eight years, and that the injury to his elbow had been sustained in a fall about two months before the present accident. It is noteworthy, in relation to the epilepsy that no morbid appearances were visible in the brain or its membranes, which could be assigned to a date earlier than the injury for which he was admitted into the hospital. The diffuse meningitis to which his death is referable, started apparently from the fractured pars petrosa of the right temporal bone. No explanation was found for the sudden strong spirt of blood from the ear on the fourth day; no wound of the carotis interna was found, and the cessation of the bleeding forbids the supposition of such source. The blood-pressure in the cerebral sinuses is known to be sufficient to expel their contents in a strong stream to the distance of more than one foot.

Soon after midday on Jan. 2d, a working jeweler, aged twenty-five, was found insensible upon the street pavement, a few yards distant from the door of his lodgings. The police brought him to the hospital; on reaching it he was still unconscious. Above the right ear was a small scalp-wound, not reaching the bone, in which no irregularity suggestive of fracture could be felt with the finger. Blood trickled from the auditory meatus. Over the outer border of the right orbit was a slight graze, and under the conjunctiva of the eye blood was extravasated. The right elbow was stiff, and it was discolored by an old bruise. Whilst being taken into the ward he vomited a coffee-ground-like fluid with some red blood-clots, and became conscious. In the afternoon his temperature in the armpit was 99° Fahr., and at 9 p. m. it had risen to 100°. The hair was cut, ice kept on the head, and thirty grains of compound jalap powder were given him.

On the 3d the bloody appearance of the fluid oozing from the right auditory meatus had changed to serous. This ear was quite deaf. He had had a restless night, and complained of intense headache, which was somewhat relieved by lying on the left side. The bowels not having acted, a compound colocynth pill was given, followed by a draught of compound infusion of senna, which purged him freely.

Next day the headache was still intense, and the temperature was high. Six leeches were placed on the right temple, and mercurial ointment was rubbed into both arms. The leeches were repeated later in the day.

On the 6th, early in the morning, the nurse was alarmed by the spouting of bright-red blood from the right auditory meatus. She stated that it was thrown out to some distance, and was not a welling-up or oozing; the amount was guessed at two ounces. The bleeding ceased spontaneously, and did not recur. Till then the serous discharge noticed on the second day had persisted. Throughout this day (the fourth after the injury) his mind wandered, but in the evening when impressively spoken to he gave opposite answers. In the night he became very delirious. At 9 p. m. the temperature was 99°. At 4:30 a. m. on the 7th he was convulsed, his face was blue, and he died.

*Necropsy ten hours later.*—Weather cold; ground covered with snow. Above the right ear was the scalp wound mentioned above, one inch long. About it, and also under and in the temporal muscle, was extravasated blood. A crack, two inches long, descended vertically through the parietal into the pars squamosa. From this a fissure crossed the pars petrosa, cutting the meatus auditorius internus, and ending in the foramen lacerum posticum basis cranii. From the meatus a second crack ran forward into the foramen lacerum medias. Other cracks joining these circumscribed a lozenge-shaped piece of the upper surface of the pars petrosa, which was quite detached. On lifting this as a lid the tympanum and labyrinth were seen plugged with blood-clot. A large blood-clot, which had its source in a large posterior branch of the middle meningeal artey, was found upon the outer surface of the dura mater in the temporal region, beneath which the cerebral convolutions were flattened. In the left side of the skull, opposite to the direct injury, under the dura mater lining the pars squamosa, was a small clot, of the size of a flattened filbert. The convolutions of this temporo-shenoidal lobe were superficially ecchymosed. The basal pia mater was congested, and its web was infiltrated with a sero-purulent, opaque, yellowish fluid. On the arachnoid, over the pons and medulla, was a thin film of exudation, and the lateral ventricles contained about four ounces of sero-purulent fluid.

CASE V. *Fall off two Steps; Copious Bleeding from the Ear and other Symptoms Strongly Suggestive of Fracture of the Base of the Skull; Recovery.*—In this case the recovery of the patient gave no opportunity of verifying the diagnosis, but the occasion of the injury—a fall on the back of the head off two steps; profuseness of the bleeding from the ear, to the extent of saturating his clothes, its continuance during several hours, and its replacement by a serous oozing; the deafness, much greater than could be fairly attributed to the rent in the membrana tympani and to the damping of the vibrations of the auditory ossicula by entangled blood-clot, and its continuance, together with the long stupor —concur in justifying the belief that here a fracture of the base of the skull crossing the left petrosal bone had occurred.

On the 20th of March, 1875, a large, heavy man, aged thirty-three, fell backwards off two steps of a doorway on to the street pavement. He was taken up insensible and brought to the hostal. On reaching it he had already so far regained consciousness that he answered when spoken to, but it was necessary to raise the voice, indeed to shout; and his reply was not always suitable to the question. When his attention was not thus roused he lay as in a deep stupor. Blood was issuing freely from his left ear, and by the amount upon his clothes it was plain that he had lost a large quantity. The most careful examination of the vertex and sides of the head failed to detect any mark of fracture or even a bruise. At nine o'clock the same evening he was still in the same stuporose state, but he occasionally tossed restlessly about and groaned. The blood still oozing from the ear was thinner and more serous. The left pupil was now widely dilated; at the time he was admitted both pupils were contracted. Not having micturated, his bladder was emptied with a catheter.

Next day oozing from the ear had ceased. The man was still very drowsy. Temperature 99.8°; pulse 80. Ice was now kept on the head. On 22nd there was extreme tenderness on and above the left mastoid process. Six leeches were put on this spot. Next day there was less tenderness and pain; less drowsy. He complained that his left ear was deaf.

On April 6th a laceration in the upper part of the membrana tympani was detected.

Three days after this note he was permitted to go home, and enjoined to report himself from time to time, which, however, he failed to do. It is believed that had he not quickly resumed work, recourse to the hospital would soon have been had for advice.

*Royal Westminster Ophthalmic Hospital.—Case of Severe Neuro-Retinitis in Both Eyes, Followed by Separation of Both Retinæ; Repeated Tappings.* (*Under the care of Mr. Macnamara.*)

For the following notes we are indebted to Mr. J. G. Mackinlay, registrar.

E. S——s, aged twenty-seven, attended as an out-patient Sept. 3rd, 1878. She had been married between five and six years, and had had three children born at full time; the eldest died in convulsions when two months old, the other two were living, and appeared healthy. She had had no miscarriages, and had always had good health. Was about six months and a half advanced in pregnancy. She stated that vision began to fail in the right eye about ten months before, and had gradually got worse. The left eye began to fail about six or eight weeks before. With the right eye she read Jaeger No. 20 with difficulty; with the left, Jaeger No. 8 at six inches; V = 20-100. On examination with ophthalmoscope the right disc could not be seen, but the vessels were visible coming out of a large dense white effusion which ran across a portion of disc obliquely, and for some distance above and below it. The left disc could be seen; vitreous hazy, and had fine movable bodies in it; no effusion of white limph on retina. No albumen in urine. Ordered mixture of per-chloride of iron with quassia.

Sept. 17th.—Condition the same. The husband attended to-day, and stated that he had a sore (for which he was cauterized) some three months before his marriage, but no history of any secondary symptoms could be obtained. The patient was ordered to continue mixture with a drachm and a half of perchloride of mercury added to each dose.

Oct. 22d.—Treatment has been continued. The upper (apparent) edge of the right disc could now be faintly seen, still there was a dense mass of white effusion over portion of disc, and a thinner extensive mass of effusion around it, and portions of vessels seen here and there plunging in and out. The left disc could not be seen; numerous small and some large floating bodies in the vitreous, and the retina was separated for about its inner fifth.

On Oct. 29th the right eye read Jaeger No. 20 at six inches, the left eye read Jaeger No. 16 at five inches. Separation of retina was noticed to-day in the right eye for about its lower and inner fifth.

The patient did not attend between Oct. 29th and Jan. 7th, 1879, but sent for her medicine. She was confined on Dec. 13th. The labor passed off well. Child was stated to be healthy; the mother was not nursing it. She stated she had an attack of inflammation in right eye a fortnight ago; the pupil was now small, irregular, and quite immovable to light. The iris was somewhat discolored, the globe slightly injected, and especially so round the ciliary region; vision was reduced to *nil*. In the left eye the pupil was large, and acted somewhat to light; vision equals perception of light.

On January 14th she was admitted. The injection of the right globe was less; the other conditions of both eyes were unaltered. Under ether, Mr. Macnamara, with a Graefe's knife, punctured sclerotic of left globe at inner and lower part, about half an inch from margin of the cornea. No fluid escaped, and the knife was inserted about a quarter of an inch further back, and fluid (not considerable in quantity) then welled up under the conjunctiva. Mr. Macnamara then tapped the right globe with Graefe's knife, puncturing the sclerotic at outer and lower part of globe about three-quarters of an inch from margin of cornea; fluid at once welled up in considerable quantity, and Mr. Macnamara then made a small slit in the conjunctiva half an inch away from the puncture, and allowed most of the fluid (which was of a pale yellow color) to escape. In both operations the knife was slightly rotated after its entrance into the globe. The eyes were padded. No sickness after the ether.

On the 15th both globes were somewhat injected; with the left eye patient could count fingers at fifteen inches and distinguish a half-crown piece.

On the 21st July patient left the hospital. She stated that she decidedly saw better, and could find her way about the ward.

On the 29th vision in right eye was unimproved, but with left eye she could read Jaeger No. 20 at six inches. The detached retina was still seen in the same position, but not extending so far up.

On Feb. 11th the left eye read Jaeger No. 20 with difficulty at five inches. Between this date and the 25th she miscarried.

On March 4th the retina was more detached in the left eye; the separation extended over almost the whole of the inner side of the fundus. She could still with difficulty make out Jaeger No. 20. She was admitted, and, under ether, Mr. Macnamara again tapped through the sclerotic with Graefe's knife and evacuated a quantity of yellow fluid, most of which is left under the conjunctiva. There was slight sickness after the ether, and next morning the globe was much injected.

On the 11th she thought her sight was not much better. The globe was still injected. She left the hospital.

On April 8th the right globe was quiet, but was getting soft. The fundus could not be seen. Vision equal to doubtful perception of light. In the left eye the retina appeared in much the same condition as before; if anything, detachment had rather increased. Vision, Jaeger No. 20 at six inches, with difficulty. Tension of left eye normal.

On May 13th the retina was still more detached in left eye; could only see fingers move. She was admitted, and Mr. Macnamara again tapped through sclerotic as before, and evacuated fluid. There was very little injection of globe after the operation. The patient left the hospital on the 17th. V = counting fingers.

On the 27th the right globe was getting very soft. With the left she saw fingers move, and retina was as much detached as ever.

The general treatment was sometimes for weeks together the perchloride of mercury in drachm and a half doses, with iron, this being occasionally omitted for a time, and small doses of strychnine given.

*National Hospital for the Paralyzed and Epileptic.—Case of Paralysis with Wasting of One Lower Extremity.* (*Under the Care of Dr. Buzzard.*)

We saw a few weeks ago the patient whose case is here described, for notes of which we are indebted to Mr. Broster, resident medieal officer. There are many points of interest in the case, especially in reference to the question of causation and treatment of perineuritis.

Agnes P——, aged thirty-six, the mother of seven children, who had never miscarried, applied at the hospital on April 30th, 1879, on account of loss of power and wasting of the right lower extremity. She had never been strong, and about five years ago suffered from a very severe attack of neuralgia of the left side of the face, which lasted five months. During the attack she lost the sense of smell, and for two years could not discern even very strong odors. In her various pregnancies the right leg always (with the exception of the last occasion) became swollen, and required an elastic bandage. After her confinements she had felt pains shooting up from the right ankle into the knee. The pain was very severe, preventing sound rest. It usually began on the second day of confinement, and lasted ten or twelve days. In October, 1878, she lost her child, and about this time felt a pain in the right knee, of a "jumping" character, shooting up the inside of the thigh. She did not know of any cause for the pain. It got worse from day to day, causing great difficulty in going up stairs. She did not notice any swelling of the knee or leg. Rest gave great relief. The pain lasted about three weeks, and was followed by a feeling of numbness. In February this year she lost much rest in consequence of having to nurse a sick child, and then felt the old pain return in the right knee and thigh, the limb always feeling very cold. Since that time the pain has got much worse, extending up the thigh to the bottom of the back, so that she has been scarcely able to move. In March she felt that the right leg was growing weak, and that she could only move it with difficulty. She noticed also that the right knee became swollen. When she was taken to the hospital she was unable to stand by herself.

The patient was a stout, healthy-looking woman with a somewhat anxious expression of face. There was very marked wasting of the muscles in front of the thigh, and loss of power in the leg, causing the foot to drop. The knee could not be lifted, and there was no power to adduction. Some tenderness was complained of on percussion over the sacrum ; there appeared to be slight synovitis of the knee-joint. The patellar tendon-reflex was absent on the right side, normal on the left, and there was no reflex on tickling the sole of the right foot. Electrical examination showed that the irritability of the muscles of the thigh and tibialis anticus group was entirely lost to the induced current. The strongest currents were only felt as "something wet" over the skin of this extremity. On the back of this thigh a pinch could be felt, though not so keenly as on the left limb. In front of the thigh it was only recognized as touch. There was nothing whatever wrong with the left lower extremity. There was no history of, or reason to suspect, syphilis. Five grains of iodide of potassium were ordered to be to taken three times a day. In three or four days she felt much relief from pain, and within a fortnight was able to move her foot slightly in bed. On June 29th it was noted that during the last few days she had suffered from pain shooting down from the knee to the ankle. There was no alteration in the electric irritability of the muscles from what had been previously noted, but strong currents now caused a slight feeling of tingling. The power of adduction had improved. On July 1st the dose of iodide was increased to ten grains, and on July 16th to twenty grains, three times daily. On July 7th she found she could limp across the room by herself. On August 16th she said that occasionally she got a twitching along the inner side of the right thigh, and aching pain (especially in wet weather) all down the right lower limb. There were no sharp, shooting pains. She was now able to stand and walk without help of any kind, and could manage to get around Queensquare. A pinch on the front and inner part of the thigh could now be felt, but not keenly as in other parts of the body ; far better, however, than when she was admitted. She recognized a touch, too, but "as though through several layers of calico." Soon after admission she had pinned some needle-work on to her knee instead of her dress, not recognizing that the pin had gone through her skin. There was still great loss of power in the anterior tibial muscles, so that voluntary dorsal flexion of the foot was impossible. The right thigh was an inch and a half, the right calf half an inch smaller than the left. She was now discharged, the treatment being continued; and when seen on Oct. 8th she could walk a mile or two, the limb had increased in size, and she was quite free from pain. The patellar tendon-reflex was still absent.

Dr. Buzzard remarked that the symptoms of the case pointed to a perineuritis of nerves of the right lower extremity. The pain between the sacrum and the limb, and the fact that both the anterior crural and the sciatic district were affected, seemed to show that the seat of inflammation was probably high up, but whether in the cavity of the pelvis or in the cauda equina could not be decided with certainty. From the history, it looked as though the nerves might have been exposed to undue pressure from the fœtus in utero, but there was probably also a proneness to inflammation of nerve-sheaths, for the woman had suffered previously from very severe facial neuralgia. The great wasting of the limb and the absence of patellar tendon-reflex might at first appear to indicate a spinal lesion, but the fact that the mobility and sensibility were equally affected in this one limb only was sufficient, he thought, to disprove this. As regards the patellar tendon reflex, a lesion in the nerve-trunk sufficiently severe to cause great muscular atrophy and cutaneous anæsthesia, would be quite as likely to occasion its disappearance as a lesion of the grey matter of the cord. The electrical reaction, too, might equally depend upon a lesion of the nerve-sheath, which practically cut the nerve off from its nutritive center in the cord. The treatment adopted was directed to a supposed rheumatic condition. He did not think that the disease was of a syphilitic origin.

*County Louth Infirmary, Ireland.—Strangulated Scrotal Hernia; Rupture of the Sac and Hæmatocele; Death. (Under the care of Dr. Hercules Macdonnell.)*

Patrick M——, aged forty-five, an active, strongly-built man, was admitted on the 22nd of August, 1879. Five years previously he noticed a swelling in his left inguinal region, which was recognized as a hernia, and for which he subsequently wore a truss. On the evening of the 20th, when jumping from a cart, he felt a snap, and immediately experienced great pain; the scrotum grew large, and the pain increased. He was seen by

Dr. Blake, of Ravensdale, the following day. An effort was made to reduce the rupture, but it proved unsuccessful.

On his admission the following morning his face wore an anxous expression. On examining the scrotum a tense, bluish, semi-fluctuating mass was seen, measuring in the lateral circumference twenty-five inches, and from the perineum to the root of the penis twenty-two inches in circumference. The bowels had not been moved for three days. There was no retention of urine. A distinct enlargement could be felt in the left inguinal region. He was given a large soap-and-water enema and a hot bath. An incision was made into the extremity of the scrotum, and twenty-one ounces of a dark fluid evacuated. This gave instant relief. The cavity was washed out with a warm, weak solution of carbolic lotion; a linseed-meal poultice, spread on tenax, was applied with oil-silk outside. One-grain doses of opium every sixth hour were ordered, and milk and lime-water in small quantities allowed. At 8 p. m. the temperature was 99.8°, and a fair share of urine had been passed since admission.

He had no sleep that night, and had next morning some pain referred to the umbilicus. No motion from bowels; temperature 99.6°; vomited some greenish matter during the night. The œdema of penis had disappeared, as well as the redness on the right side of scrotum, but the left was very much enlarged. Some blackish matter came away on the poultice. Hernial neck now well defined; slight gurgling heard on manipulation. During the day no flatus or fæces passed; the vomiting was frequent and large in quantity, but not stercoraceous. In the afternoon he was given a long bath at 100° F. Half a grain of morphia was injected subcutaneously, and the operation for the division of the stricture was commenced, ether spray being used. No difficulty was experienced in reaching the sac, which was found very much thickened. On opening this the intestine was found to be bluish and congested, but not gangrenous. The stricture was relieved, but considerable and unaccountable difficulty was found in returning the intestine, of which there were three large loops down. The patient was removed to a warmed bed, some slight timulant given, and warm jars applied to the feet. Antiseptic dressings were applied. At 11 o'clock the patient complained of feeling sick. Some flatus escaped, and during the act of vomiting he fell back in the bed, dead.

He was quite cheerful a few moments before, and complained of no pain.

*Necropsy twelve hours afterwards.*—Rigor mortis well marked. Penis and scrotum discolored, also left iliac region. On removing the silver sutures and prolonging the incision downwards, laying open the scrotum from apex to base, about a teaspoonful of dark-colored fluid escaped. The sac of the hernia was adherent to the tissues for a great part of its extent. The testicle was smaller and softer than normal. At the lower and back part of the sac, where it began to be reflected forwards, was a rent, extending for about an inch in a horizontal direction, of a lacerated description; this was sufficient to account for the hæmatocele, and also for the difficulty experienced in returning the hernia when the structure had been fully divided, a knuckle of intestine having passed through this rent in the sac. The filling up of the scrotum took place very quickly, and immediately on receipt of the injury.

The presence of a hæmatocele with scrotal hernia is sufficiently rare to merit notice; but the cause—viz , rupture of the sac from a violent jump—is in itself such a rare injury that a case verified by post-mortem examination is worthy of being recorded.

*Tunbridge Wells Infirmary.—Case of Membranous Laryngitis; Tracheotomy; Recovery.* (*Under the care of Mr. Rix.*)

For the following notes we are indebted to Mr. John Footner, F. R. C. S., house-surgeon.

E. D——, aged five years, was admitted May 13th, 1879, suffering from stridulous dyspnœa and sore throat, with a moderate amount of fever.

Her sister had been taken ill with similar symptoms two days previously, and died the second day of the disease. On admission, the patient was seen to be a rosy-cheeked child, very healthy looking and well nourished. Her pharynx and palate were congested, and tonsils much enlarged. There was no trace of false membrane anywhere visible. Respiration was performed with difficulty, stridulous, and much accelerated, 48 per minute. Pulse 168; temperature 100° Fahr. There was no cyanosis.

She was placed in a room the air of which was moistened and warmed by steam to a temperature ranging from 70° to 76° Fahr., which was maintained throughout her illness.

Mr. Rix ordered half a grain of calomel to be taken every four hours, and also a mixture containing antimony and ipecacuanha. No improvement in the patient's state took place, and on the following day, as the dyspnœa had become very urgent, and she was much cyanosed, chloroform was administered, and tracheotomy above the isthmus of the thyroid body was performed by Mr. Rix, and a silver tube introduced. Immediately after the operation the dyspnœa was greatly relieved, and the cyanosis gradually disappeared. A good deal of mucus was expelled through the tube just after the operation, but no trace of any false membrane. Patient passed a fairly quiet night, but coughed up a good deal of mucus through tube. Next day the respiration was much quieter than before, but still it was much accelerated, 34 per minute. She took liquid nourishment well. Antimony discontinued.

On the 17th she coughed up some small pieces of tough leathery false membrane through tube. Cough rather frequent. Lung sounds, clear. Small pieces of false membrane were coughed up during the two following days.

On the 18th the calomel was ordered to be given every eight hours, and on the 21st it was discontinued. The wound round tube looked sloughy and very unhealthy. The child could not swallow perfectly; on attempting to swallow some milk most of it regurgitated through tube and anterior nares. Respiration still very quick, 44 per minute. The lung sounds were perfectly healthy. She had rapidly become pale and emaciated.

On the 22nd the tube was removed in the morning, but it was found necessary to replace it at night owing to increased dyspnœa. While the tube was out, the dysphagia still remained, although the aperture in the neck was closed by the finger during deglutition.

On the 24th a small quantity of albumen (1 in 7) appeared in the urine. The temperature, which up to this time had fluctuated between 101° and 102° F., sank to 100°; respiration 30 per minute. The patient was allowed a boiled egg and bread and butter, which she swallowed well.

On the 26th temperature was normal, and the patient was much better. The urine still contained a trace of albumen. Deglutition was performed more easily, and rapidly improved after this date.

On the 29th the tube was removed. From this time the patient began to regain her health and strength. The steam was gradually dispensed with, and she was allowed solid food.

On January 7th she got up and was removed into a large ward amongst the other patients.

Her convalescence was retarded by a mild attack of tonsillitis and bronchitis, which subsided under appropriate treatment.

She was discharged from the infirmary cured on July 7th.

*Remarks.*—This case presents most of the symptoms of diphtheria—a disease from which, as is well known, tracheotomy does not, as a rule, afford much hope of recovery. The persistent high temperature, ranging from 101° to 102° during a period of thirteen days; the presence of albumen in the urine, though only during a short period of four days, and the dysphagia; the rapid change from a fat, rosy-looking child to a pale and emaciated one (very probably caused by the calomel); the fact of her sister having been taken ill two days previously with similar symptoms, and the rapidly fatal termination; the expulsion of pieces of false membrane through the tube—all point to diphtheria; but it must be added that there was no enlargement of the glands at angle of jaw, nor was any false membrane visible *in situ.* Tracheotomy was performed early in this case, which, perhaps, may have contributed a good deal towards the patient's recovery.

(From the *London Lancet.*)

---

WE hope to present to our readers in our next issue, notices of the medical college commencements, which have been and are still to be held in this city, with lists of the graduates of each. The proceedings of the various alumni associations, and of the Cook County Hospital Association, will also be then reported in full.

